# Risk stratification in autoimmune cholestatic liver diseases: Opportunities for clinicians and trialists

**DOI:** 10.1002/hep.28128

**Published:** 2015-11-26

**Authors:** Palak J. Trivedi, Christophe Corpechot, Albert Pares, Gideon M. Hirschfield

**Affiliations:** ^1^ National Institute for Health Research (NIHR), Birmingham Liver Biomedical Research Unit (BRU), and Center for Liver ResearchUniversity of BirminghamBirminghamUnited Kingdom; ^2^ National Reference Center for Inflammatory Diseases of the Biliary Tract (MIVB), Rare Liver Diseases Health Network (FILFOIE), Saint‐Antoine HospitalAssistance Publique‐Hôpitaux de Paris (APHP)ParisFrance; ^3^ Liver Unit, Hospital Clínic, CIBERehd, IDIBAPSUniversity of BarcelonaBarcelonaSpain

## Abstract

Primary biliary cirrhosis (PBC) and primary sclerosing cholangitis (PSC) are infrequent autoimmune cholestatic liver diseases, that disproportionate to their incidence and prevalence, remain very important causes of morbidity and mortality for patients with liver disease. Mechanistic insights spanning genetic risks and biological pathways to liver injury and fibrosis have led to a renewed interest in developing therapies beyond ursodeoxycholic acid that are aimed at both slowing disease course and improving quality of life. International cohort studies have facilitated a much greater understanding of disease heterogeneity, and in so doing highlight the opportunity to provide patients with a more individualized assessment of their risk of progressive liver disease, based on clinical, laboratory, or imaging findings. This has led to a new approach to patient care that focuses on risk stratification (both high and low risk); and furthermore allows such stratification tools to help identify patient subgroups at greatest potential benefit from inclusion in clinical trials. In this article, we review the applicability and validity of risk stratification in autoimmune cholestatic liver disease, highlighting strengths and weaknesses of current and emergent approaches. (Hepatology 2016;63:644–659)

**Abbreviations**
AEs: adverse events
ALP: alkaline phosphatase
AMA: anti‐mitochondrial antibody
ANA: anti‐nuclear antibody
AST: aspartate aminotransferase
APRI: AST/platelet ratio
AUROC: area under the receiver operator curve
CCA: cholangiocarcinoma
DS: dominant strictures
EFS: event‐free survival
ELF: enhanced liver fibrosis score
ERC: endoscopic retrograde cholangiography
GEVs: gastroesophageal varices
HCC: hepatocellular carcinoma
IBD: inflammatory bowel disease
IgG4: immunoglobulin G subclass 4
LSM: liver stiffness measurement
LT: liver transplantation
MRC: magnetic resonance cholangiography
PBC: primary biliary cirrhosis
PH: portal hypertension
PSC: primary sclerosing cholangitis
sdPSC: small duct primary sclerosing cholangitis
SMR: standardized mortality ratio
TFS: transplant‐free survival
UDCA: ursodeoxycholic acid
ULN: upper limit of normal
VCTE: vibration controlled transient elastography

Primary biliary cirrhosis (PBC) and primary sclerosing cholangitis (PSC) are chronic autoimmune cholestatic liver diseases, for which clinical outcome is largely dictated by development of cirrhosis, portal hypertension (PH), and variable predisposition to malignancy.^1^, ^2^, ^3^, ^4^ Rates of clinical progression vary, and accurately identifying disease course is of critical importance to patients, clinicians, as well as those committed to developing new, effective and affordable treatments.^5^ Patients seek reassurance and guidance as to their own prognosis, and clinicians wish to confidently recognize those at highest risk of poor outcomes as equally as they strive to reassure individuals with good prognosis. Partnerships with industry are essential to drug development; and collectively all those involved in clinical trial design, recruitment and analysis wish to understand unmet need and conduct studies of new therapies as carefully constructed interventions that deliver Specific, Measurable, Achievable, Relevant and Time‐cost limited outputs. Such ventures seek to “de‐risk” drug development pathways where possible, but maximize opportunity to advance therapy for patient benefit in a timely way.

Herein, we present an appraisal of existing parameters that stratify individuals with PBC and PSC, before examining the effectiveness and applicability of more incipient classification systems (Fig. 1). The strengths and weaknesses of various approaches are highlighted specifically throughout, as well more generally with regard to study design (Table 1).

**Table 1:** Common Pitfalls in Risk Stratification Studies

Precedents	Apprehensions
**Well recognized; frequently addressed**	
Inadequate follow‐up time	• Restricts assessment to late‐stage events • Poorly predictive of at‐risk groups from disease outset
Single‐center studies	• Lack of independent, external validation • May not be generalizable
Small sample size	• Inadequate statistical power • Increased type 1 error
**Well recognized; infrequently addressed**	
Representation limited to retrospective data collection	• Incomplete data • Selection bias and confounding factors • Reduces statistical power
Extrapolation of risk stratifiers beyond original intended endpoint/time point	• Increased type 2 error
Tertiary‐center‐restricted studies	• Recruitment/referral bias; not necessarily representative of the disease globally
Logistic regression (odds ratios; ORs) applied for clinical endpoints that are not time‐constrained	• Time‐variable events (e.g., death, transplantation) necessitate Cox regression (hazard ratios; HRs), or equivalent
“Time‐dependent” covariates modeled as “time‐constant”	• Inadequate representation of chronal displacement between potential risk factors and the measured endpoint
**Poorly recognized; infrequently addressed**	
Predictive utility of a continuous variable not proven prior to applying to dichotomization	• Subjugation of variability • Increased type 1 error • Conceals nonlinearity between covariates and clinical outcomes
Independently predictive covariates deemed additive	• Incorrect classification of risk through weaker stratifiers
Incorrect estimation of median EFS times	• Incorrect approach: ‐ Calculated median: midpoint of all patients' follow‐up times • Correct approach: ‐ Actuarial median: time at which 50% of the cohort meets the clinical endpoint according to Kaplan‐Meier estimates
Contrary inferences	• Favorable clinical outcomes suggested for low‐risk groups stratified according to dichotomous variables, despite EFS dwarfing that of a matched control population

**Figure 1:**
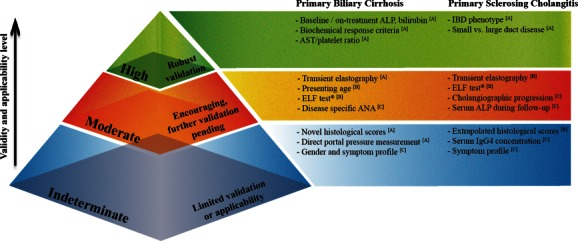
Approaches to risk stratification in autoimmune cholestatic liver disease. The presented infographic illustrates the authors' ranking with regard to currently available prognostic models and scoring systems, ordered dependent on predictive performance, validation status, and routine clinical applicability. For instance, biochemical response criteria represent the most robust discrimination method of at‐risk populations in PBC and can be assessed noninvasively by clinically acceptable means. However, liver histology is perhaps the most biologically representative index of disease progression (PBC and PSC), yet routine, ongoing assessment through serial liver biopsies clearly unacceptable in routine clinical practice. Nevertheless, application of robust noninvasive surrogates holds promise, (particularly for transient elastography, which as a fibrosis indicator is very well substantiated in PBC) and may be extrapolated to forecasting clinical outcomes. Further validation of these modalities as independent and, more so, additive predictors is eagerly awaited, particularly to discriminate severity versus stage of disease. Conversely, the emergence of serum IgG4 and serum ALP as putative risk stratifiers in PSC are not supported by well‐controlled or high‐quality validation, and studies incorporating assessment as continuous variables with inclusive control populations are urgently commanded. Levels of evidence for each stratagem are indicated in superscript according to the recently revised Grading of Recommendations Assessment, Development and Evaluation criteria (GRADE) criteria for assessment of prognosis (high = A; intermediate = B; low = C; very low = D) (Supporting Table 1 78).

## Clinical History and Phenotypes

The full appreciation of the breadth of PBC as a disease has evolved as awareness has risen, particularly given widespread access to anti‐mitochondrial antibody (AMA) testing, reactivity of which in the presence of cholestasis facilitates robust and timely patient identification without need for histological confirmation.^6^, ^7^ PBC is increasingly identified at an earlier precirrhotic stage,^8^ and well‐conducted multicenter cohort studies have aided in the recognition of variant presentations (Table 2), including male patients and women age <50 years.^9^ Ursodeoxycholic acid (UDCA) is the only approved therapy, with diminished disease progression evident in treated patients and significantly improved 10‐year transplant‐free survival (78% vs. 66%; *P <* 0.001).^3^, ^8^, ^10^, ^11^, ^12^ Pooled survival indices nevertheless remain lower than age‐ and sex‐matched control populations.^10^, ^11^, ^12^

**Table 2:** Variant Presentations

A. PBC	
Phenotypic Variant (% of Patient Population)	Prognostic Implication
Male sex 2, 9, 32 (∼5%‐10%)	• Older age at diagnosis relative to women (60 vs. 55 years; *P* < 0.001) • Greater frequency of nonresponse (63% vs. 76%; *P* < 0.001) ‐ Likely attributable to more advanced baseline disease • Increased HCC risk in biochemical nonresponders, as well as patients with cirrhosis
Young presenting age^9^, ^32^ (∼25%)	• Biochemical response rate in women <40 years old at diagnosis is less than 50%
AMA negative^8^, ^9^, ^28^ (∼5%‐10%)	• Clinical course identical to AMA‐positive PBC
Intractable pruritus^12^, ^17^ (dynamic frequency reported)	• Consider referral for clinical trials specifically targeting pruritus • Rarely can be severe enough to merit transplantation as the solitary indication
Overlap^a^ with autoimmune hepatitis (AIH)^14^ (∼3%‐20%; diagnostic criteria inconsistent)	• Severity of interface hepatitis predictive of biochemical nonresponse ‐ Unclear whether biochemical response criteria predict prognosis in overlap • Inconclusive data regarding clinical outcome relative to PBC alone

B. PSC	
Phenotypic Variant (% of Patient Population)	Prognostic Implication
Young presenting age^14^ (frequency unclear—lack of consensus on disease nomenclature between children and adult patients)	• Under 16 years more commonly present with an inflammatory phenotype • Speculated to endure a more rapidly progressive course (unconfirmed)
Small duct disease^13^, ^62^, ^63^, ^64^ (10%‐15%)	• Less often symptomatic (30% vs. 53%; *P <* 0.01) • Disease progression infrequent relative to classical PSC ‐ Transplantation rate ∼10% ‐ TFS relative to classical form: 29 vs. 17 years; *P* = 0.04 • Increased risk of CCA not evident in small duct disease • Up to 25% may progress to large duct disease over ∼7 years
Dominant strictures^50^, ^60^ (12%‐60%)	• Strictly defined on ERC criteria • Increased mortality largely attributed to inability in differentiating benign from malignant lesions • Reduced TFS (particularly if coexists with colitis) ‐ 25% vs. 73% over 20 years; *P* = 0.011
Coexisting Crohn's disease^75^ (13%; one study)	• 50% female • 22% have small duct PSC—consequently exhibit improved LT‐free survival • Impact of small intestinal vs. colonic Crohn's not yet discerned
Elevated serum IgG4 levels^46^, ^47^, ^48^, ^49^ (9%‐15%)	• More significant elevations in serum liver biochemistry/Mayo score • Impact on clinical outcome unclear
Overlap^a^ with autoimmune hepatitis (AIH)^14^ (∼1%‐53%; diagnostic criteria inconsistent)	• 50% of children diagnosed with AIH age <16 years “evolve” into PSC • Inconclusive data regarding clinical outcome relative to PSC alone • Frequency of coexisting IBD similar (PSC likely dominant disease process)

a The prevalence of overlap is difficult to ascertain because of publication bias, variable definitions, and considerable heterogeneity between syndrome designations. Moreover, the limitations of applying surrogates of outcome to settings distinct from which they were originally intended must be recognized (covered elsewhere^14^). Given small numbers of patients comprising few nonrandomized, nonblinded studies, evidenced‐based risk‐stratification centerd on the relative presence/absence of overlap features is not possible currently and worthy of prospective multicenter collaborative investigation.

Modeling the clinical course of PSC, in contrast to PBC, is far more testing, perhaps inevitably so given a lower incidence and absence of a defined serological marker. This is paralleled by a clinical phenotype driven by variable, unpredictable consequences related to chronic inflammation, fibrosis and neoplasia of medium‐ to large‐sized bile ducts. In the largest population‐based study to date (n = 590), disease was validated as being male predominant (∼60%), with a median age at diagnosis of ∼40 years.^13^ However, PSC can develop at any age, with younger patients frequently manifesting a hepatitic presentation.^14^ Associations with inflammatory bowel disease (IBD) are well recognized and ∼70% of PSC patients have a history of colitis, which confers a 5‐fold greater risk of colonic cancer relative to IBD alone, as well as increased susceptibility to cholangiocarcinoma (CCA) independent of liver disease stage. PSC portends a standardized mortality ratio (SMR) more than 4‐fold that of a matched control population, although there is discrepancy between event‐free survival (EFS) times across transplant centers versus true population‐based cohorts (median, 13.2 vs. 21.3 years; *P <* 0.001^13^). Population‐level data thus highlight significant challenges to prognostic modeling and unmask the inadequate phenotypic representation of early‐stage disease and inherent selection bias with tertiary‐center‐restricted reporting.

### Symptom Complex

Pruritus and fatigue are frequent symptoms associated with cholestasis^15^ and approximately 60% of patients with PBC are asymptomatic at diagnosis, with as few as 5% remaining symptom free over time.^16^ The prognostic importance of fatigue in PBC is contentious, but concern is perhaps best highlighted in the prospective cohort study from Jones et al. (n *=* 136),^12^ wherein transplant‐free survival (TFS) was significantly shorter among fatigued patients relative to nonfatigued, disease‐matched controls (56% vs. 74%; *P* < 0.0001), independent of UDCA provision. Although a consensus biological explanation for fatigue is lacking, presenting age and sex heavily influence the clinical phenotype, with young women (a group failing UDCA therapy more commonly) having the greatest symptom burden.^9^, ^17^ However, there is no evidence that symptomatic presentations impart additional discriminatory value to existing risk‐prediction models.

Symptomatic presentations in PSC similarly vary (36%‐56%), with over 20% developing symptoms *de novo* during follow‐up.^18^, ^19^, ^20^ Relapsing‐remitting episodes of acute cholangitis are a frequent concern; and data from several cohorts suggest symptomatic presentations carry poorer TFS and malignancy‐free survival.^18^, ^20^ One third of CCA are diagnosed within the first year of PSC presentation (annual incidence thereafter: 0.5%‐1.5%; lifetime risk: 7%‐15%),^13^, ^18^ and patients often report abdominal pain preceding diagnosis, particularly those with a prolonged history of IBD (>1 year).^18^, ^21^

## Biochemical Response Criteria in PBC

Serum bilirubin is well established as a predictor of outcome and incorporated into several prognostic scoring systems.^22^, ^23^ However, “time‐constrained” models, such as the Mayo score, which include bilirubin together with other markers of cirrhosis, are limited to prediction of short‐term survival (<2 years) in relatively late‐stage disease. A potentially more applicable surrogate is serum alkaline phosphatase (ALP); and in the largest ever meta‐analysis of individual patient data (n *=* 4,845), a near log‐linear relationship was illustrated between ALP and subsequent risk of transplantation/death across several time points.^8^ This study demonstrated that ALP bestows prognostic information early in the clinical course, incremental to the predictive power of bilirubin and independent of follow‐up time, presenting age, sex, disease stage, and treatment status.

To this effect, several studies illustrate strong associations between percentage reduction or absolute decreases/normalization in serum ALP (in isolation or combination with other biochemical covariates) and significantly improved clinical outcome.^10^, ^11^, ^24^, ^25^ Indeed, the majority who successfully attain predefined biochemical thresholds 1‐2 years after UDCA treatment (13‐15 mg/kg/day) experience survival patterns akin to that of an age‐ and sex‐matched control population (Table 3A). All response criteria have been independently and externally validated, with Paris I capturing the greatest breadth of biochemical changes. Furthermore, there is clear, negative prognostic impact of biochemical nonresponse on future hepatocellular carcinoma (HCC) risk in PBC patients, independently and additive to the effects posed by male sex and advanced baseline disease stage.^2^

**Table 3:** Biochemical Response in PBC

A. Established Models Derived From Biochemical/Laboratory Parameters (2006‐2010)				
Proposed Response Criteria (Attainment Rate)	Derivation Cohort; No. of Patients	Clinical Event Rate; R vs. NR; Log Rank (Interval)	Validation Series; No. of Patients	Comments
**Barcelona** 11 >40% decrease in ALP (or normalization) from baseline (achieved by 61% of patients)	n = 192; single center	3% vs. 17%; *P* < 0.01 (median follow‐uo 7.5 years)	n = 2,353; multicenter (N)^9^ n = 1,015; multicenter (I)^28^	Delta change in ALP less predictive than absolute reduction in subsequent series^8^
**Paris I** 10 ALP <3× ULN, and AST <2× ULN, and bilirubin ≤1 mg/dL (achieved by 61% of patients)	n = 292; single center	10% vs. 49%; *P* < 0.001 (at 10 years)	n = 2,353; multicenter (N)^9^ n = 1,015; multicenter (I)^28^	Nonresponse also predictive of HCC risk^2^ Most robustly validated of all criteria
**Rotterdam** 76 Albumin and bilirubin normalization (achieved by 76% of patients)	n = 311; multicenter (N)	19% vs. 44%; *P* < 0.001 (at 10 years)	n = 1,015; multicenter (I)^28^	Nonresponse also predictive of HCC risk^2^ Limited applicability in early‐stage disease
**Toronto** 25 ALP <1.67× ULN	n = 69; single center	N/A	n = 2,353; multicenter (N)^9^ n = 1,015; multicenter (I)^28^	Nonresponse also predictive of HCC risk^2^ Endpoint in the derivation study was histological progression, whereas association with clinical outcome verified in subsequent validation series.

B. Modification of Existing Response Criteria (2011‐2013)				
Proposed Response Criteria	Derivation Cohort; No. of Patients	Clinical Event Rate; R vs. NR; Log Rank (Interval)	Validation Series; No. of Patients	Comments
**Paris II** 24—early stage PBC only ALP ≤1.5× ULN, AST ≤1.5× ULN, andbilirubin ≤1 mg/dL (achieved by 48% of patients)	n = 165; single center	0% vs. 13%; *P* < 0.001 (mean follow‐up 7 years)	n = 2,353; multicenter (N)^9^ n = 1,015; multicenter (I)^28^	Externally validated; Nonresponse also predictive of HCC risk^2^ Concern over modest sensitivity (50%) and low NPV (13%) may incorrectly stratify high‐risk patients
**Early biochemical response** 27 Paris; Barcelona; Toronto met at 6 months.	n = *187*; single center	See comments	Not yet validated	Biochemical response rates (Paris I; 71% vs. 70%) and respective PPV (0.90 vs. 0.91)/NPV (0.45 vs. 0.47) of future clinical events identical at 6 vs. 12 months

C. Optimization of Previous Response Criteria (2013‐2015)				
Proposed Response Criteria	Derivation Cohort; No. of Patients	Follow‐up Interval (Years)	Validation Series; No. of Patients	Net Reclassification Index (NRI) (%)
**Biochemical response + APRI‐r1 ≤0.54** a, 28	n = 352; single‐center	Median: 7	n = 446; multicenter (I)^28^	20
**UK‐PBC risk score** b, 34 Prognostic index comprising bilirubin, ALP ratio, AST, ALT albumin, and platelet count	n = 1,916; multicenter (N)	Median: 7	n = 1,249; multicenter (N)^34^	Not specified
**GLOBE score**; www.globalpbc.com 35 Prognostic index comprising patient age, bilirubin, ALP ratio, albumin, and platelet count	n = 2,488; multicenter (I)	Median: 8	n = 1,631; multicenter (I)^35^	25‐35

Several response criteria are proposed in PBC, wherein LT‐free survival akin to that of a matched population is predicted after attainment of well‐defined parameters; most often applied at 1‐2 years after UDCA treatment/PBC diagnosis. Although the optimum cutpoint for serum ALP is difficult to define, it is apparent that absolute levels during follow‐up predicts outcome with higher accuracy relative to percentage decrease.

Event rates in responders (R) vs. nonresponders (NR) are provided for principle studies in (A). Modifications to existing criteria; specifically, targeting patients with early‐stage disease, as well as 6‐ vs. 12‐month biochemical response have been attempted, although the latter approach is awaiting validation (B). Improvements of previous response criteria are being attempted, with examples provided for current studies (C). Single‐ and multicenter national (N) and international (I) studies are denoted accordingly.

a Application of APRI score at 1 year (APRI‐r1) to all preexisting biochemical criteria has been shown to improve predictive performance.

b Endpoint in the UK‐PBC study was transplantation, or liver‐related (as oppose to all‐cause) mortality.

Abbreviations: ALT, alanine aminotransferase; N/A, not applicable; PPV, positive predictive value; NPV, negative predictive value.

Although a small proportion of PBC patients with early‐stage disease meet response criteria free of therapy,^26^ this represents an understudied population, and presently, it is not possible to identify individuals likely to endure a good prognosis regardless of intervention. Inversely, paradigms reliant on waiting 1 year for therapeutic evaluation may leave high‐risk patients (future nonresponders) on a medical treatment lacking benefit and reduce impact of second‐line therapy because of delayed initiation. In this regard, a prospective study from China suggests that attainment rates, as well as predictive value, is identical when biochemical response is assessed at 6 versus 12 months (Table 3B),^27^ but this needs validation.

### Demographic Variations

Population‐level and international multicenter studies have substantiated the predictive performance of biochemical response criteria, independently of disease stage and UDCA exposure.^9^, ^28^ Perhaps most notable is the UK‐PBC study (n *=* 2,353), which not only recognized an increasing prevalence of younger presenting women (25% age <50), but also an inverse correlation of patient age and likelihood of meeting biochemical response.^9^ Attainment rates were reportedly ≤50% in women age below 40 and echo results of an earlier, single‐center study wherein age <55 years conferred poorer relative survival compared to matched controls (SMR, 7.4).^29^ Younger women often present with more pronounced elevations in serum ALP^17^ but frequently fail therapy owing to transaminase elevations,^9^ possibly reflecting a more hepatitic phenotype. This is noteworthy given that the degree of interface activity is recognized to influence disease progression in PBC.^10^, ^14^, ^30^, ^31^ The impact of presenting age was less apparent in men,^9^ who, despite being older at diagnosis, exhibited greater frequency of nonresponse overall, possibly reflecting more advanced baseline fibrosis at presentation.^32^

The strong influence of presenting age may allow more timely stratification of at‐risk groups (preceding assessment of 12‐month biochemical response), who, because of a relatively poor predicted survival, would be potentially eligible for early clinical trial entry. However, the more opportune recognition of at‐risk individuals must ensure that low‐risk patients are not over treated,^27^ particularly given that 50% of all patients under 50 do indeed meet current biochemical response criteria on UDCA.^9^

### Optimization of Criteria

Existing biochemical response criteria remain to be refined, with a subgroup of responders still at risk of developing adverse events. There is evidence that reduction in hepatic venoportal gradient whereas on UDCA treatment associates with improved TFS in PBC, stratifying through a 20% gradient decline over 2 years.^3^ Conversely, the presence of gastroesophageal varices (GEVs) is a poor prognostic factor^4^; and given that PH can develop in the absence of cirrhosis secondary to presinusoidal resistance, several algorithms for prediction of GEVs are proposed. Although advocated for guiding variceal surveillance, such models carry preselection bias, given that study populations from which they derive were included after endoscopy referral. Moreover, no current strategy allows noninvasive discrimination of clinically significant PH.

With regard to patient survival, performance characteristics of the aspartate aminotransferase (AST)/platelet ratio index (APRI) have been ascertained given ability to infer not only PH, but also fibrosis.^3^, ^28^ When applied at baseline or at 1 year, APRI was identified as an independent predictor of TFS across a tertiary center population (n = 386), with a discriminatory cutpoint of 0.54 externally validated in three international cohorts.^28^, ^33^ Moreover, 1‐year APRI identified the subgroup at risk of disease progression and earlier mortality despite successful attainment of biochemical response (Table 3C), indicating independent and additive prognostic information to existing criteria.^28^, ^34^, ^35^

Newer, highly complex, and robust computational algorithms incorporating facets of APRI in addition to conventional biochemical response parameters have recently been published. These scoring systems derive from large, multicenter cohorts as part of UK‐PBC as well as the Global PBC Study Group^34^, ^35^ and convey probability of TFS on a continuous, as opposed to dichotomous, scale (area under the receiver operator curve [AUROC]: >0.9). In addition to being internally validated, the latter in particular has been compared against a healthy age‐ and sex‐matched control population, demonstrating comparable prognostic performance to Paris‐I + APRI.^35^ However, it remains uncertain above what point patients will be deemed high risk enough for clinical trial stratification, how the modifier effects of UDCA on risk score will influence outcome (delta change), and which additional stratifiers will continue to retain independent clinical impact.

## Can Biochemical Surrogates Be Extrapolated to PSC?

Serum bilirubin is inherent to many historic PSC prognostic models, including the disease‐specific Mayo score.^36^ Despite widespread application, the series from which the latter derives antedates modern management of variceal bleeding and receives further criticism given inability to foreshadow adverse events (AEs) in previous clinical trials.^37^ Although a persistently elevated bilirubin for >3 months incites concern for hepatobiliary malignancy,^18^ levels have a propensity to fluctuate with flares of cholangitis and potentially influenced by biliary interventions.

There is no proven survival advantage, or reduction in hepatobiliary/colorectal malignancy risk for PSC patients receiving UDCA, and an increased predisposition toward AEs well documented with high dosages (28‐30 mg/kg/day).^1^, ^5^ Several groups have nevertheless attempted construction of “ALP‐based” biochemical response criteria (Table 4),^38^, ^39^, ^40^, ^41^, ^42^, ^43^ but ultimately, each has failed cross‐validation at the originally conceived time points. For instance, the 1.5× the upper limit of normal (ULN) cutpoint proved discriminatory at 2 years in the Oxford cohort (irrespective of UDCA receipt^40^), but was only predictive when applied at 6 and 12 months in the Heidelberg and national UK series, respectively. Moreover, in only one published study has the predictive value of ALP as a continuous variable been confirmed before establishing utility through dichotomization^43^; however, full statistical methodology was not presented and clinical endpoints incorrectly assessed as time‐constrained events.

**Table 4:** Proposed ALP Thresholds in PSC

Proposed Criteria (Attainment rate)	Derivation Cohort; No. of Patients	Clinical Event Rate; R vs. NR (Interval)	Endpoints Tested	Apprehensions
**Rochester** a, 38 ALP normalization at any point (median attainment time: 1 year) (Achieved by 40% of patients)	n=87; single‐center	14% vs. 33%; *P*=0.02 (median follow‐up: 7.3 yrs)	Death, LT, CCA	• Small number of patients 20% of UDCA‐treated individuals reached clinical endpoint despite normal serum ALP
**Oxford** 40 ALP <1.5× ULN at 2 years (achieved by 40% of patients)	n=139; single‐center	6% vs. 38%; *P* < 0.0001 (median follow‐up 10 years)	Decompensation, Death, LT, CCA	• Tested in Heidelberg study at 6 months^39^ and UK‐PSC multicenter study at 1‐year time point^41^ • ALP threshold not successfully validated at original 2‐year time point
**Scandinavian multicenter** 42 ALP >40% decline from baseline or normal at 1 year. (achieved by 40% of patients)	n=195; multicenter (I)	Rate not specified; Difference between groups: *P* < 0.001	Death, LT, CCA	• Not successfully validated^39^
**Heidelberg** 39 ALP <1.5× ULN, or ALP ≥50% decline, or ALP normal 6 months from baseline (achieved by 51% of patients—any above)	n=185; single‐center	13% vs. 49%; *P* < 0.05 (median follow‐up: 10 years)	Death, LT, CCA	• Clinical event rate not significantly different between groups when ALP <1.5× ULN threshold applied (in isolation) at 1 year
**UK‐PSC** b, 41 Criteria (1): ALP <1.5× ULN at 1 year Criteria (2): ALP <2.0× ULN at 2 years (attainment rates not yet available)	n=1,200; multicenter (N)	Rate not specified; Significant difference between groups: (1) *P* < 0.001 and (2) *P*=0.015	LT only	• Threshold of ALP <1.5× ULN did not prove discriminatory when applied at 2 years

Emerging biochemical response criteria in PSC patients based on varying thresholds of serum ALP applied 6‐24 months after diagnosis. Attainment of these criteria is purported to infer significantly improved clinical outcome in the individual cohorts studied, although comparisons to matched control population are yet to be drawn, and none of the inclusive studies have assessed serum ALP as a continuous variable before application of presented cutpoints.

a Predefined time point not specified.

b Full results yet to be published.

Systematic efforts to validate the prognostic utility of serum ALP in PSC therefore remain in their infancy, and none of the studies thus far incorporate a comparator control group. Therefore, it is difficult to infer what an improved serum ALP truly means, given that “PSC biochemical responders” may still benefit from trials of new therapy if survival significantly deviates from the healthy population. Spontaneous normalization has been reported in up to ∼40% of patients;^38^ and whereas this may indicate a slowly progressive form of disease, based on available evidence ALP cannot be recommended as a stand‐alone stratifier of risk in PSC.

## Immunoserological Indices and Coexisting Autoimmunity

### PBC‐Specific Anti‐Nuclear Antibodies

Unlike AMA, which holds no prognostic value,^8^, ^9^, ^28^ there exist several anti‐nuclear antibody (ANA) subtypes that may associate with adverse clinical outcome in PBC. Baseline anti‐gp210 reactivity imparts over a 6‐fold risk of progression to liver failure/transplantation^44^ and although neither independent nor additive to biochemical response^12^, ^30^ may assist in the earlier, prospective identification of high‐risk patients.^27^, ^44^ Anti‐centromere antibodies similarly associate with PH,^44^ although more often present in autoimmune connective tissue disease. Extrahepatic autoimmunity develops in ∼60% of PBC patients; however, impact on liver‐related outcomes is not readily apparent.^45^

### Serum Immunoglobulin G Subclass 4 in PSC

Between 9% and 15% of PSC patients have raised serum immunoglobulin subclass 4 (IgG4) values,^46^, ^47^, ^48^, ^49^ and at least three separate studies support clinical distinctions based on elevations; those having higher than normal values (>1.4 g/L) exhibiting greater derangements in liver biochemistry.^46^, ^47^, ^48^ One group identified shorter median time to transplantation in patients harboring elevated serum IgG4,^48^ although this observation has repeatedly failed replication across several international centers.^49^ Therefore, the stratifying properties of serum IgG4 in PSC remain unsubstantiated and require further evaluation.

### Impact of Colitis in PSC

Several historic studies suggest that the presence of colitis influences liver disease progression. However, many were flawed given their assessment of IBD as a time‐fixed covariate; and the chronological displacement of disease presence and activity between gut and liver manifestations impart significant difficulties in examining colitis as a risk stratifier. Nevertheless, in a prospective follow‐up of nearly 200 PSC patients, all hepatobiliary malignancies were observed to develop on a background of concurrent colitis, with no cancers in the absence of IBD.^50^ Moreover, TFS independent of CCA was also significantly different between groups (23% vs. 80%; *P* = 0.045). The negative prognostic impact of colitis on liver‐related outcomes has since been confirmed in a large Dutch PSC cohort (n *=* 161) as well as two population‐based series.^13^, ^51^, ^52^, ^53^

## Cholangiographic Stratification in PSC

Several cholangiographic prognostic models derived from endoscopic retrograde cholangiographic (ERC) appearances have been proposed^54^; however, diagnostic paradigms have evolved and no correlation between severity of ductal involvement and survival through two‐dimensional magnetic resonance cholangiography (MRC) was demonstrated. Nevertheless, a promising study utilizing annual three‐dimensional MRC to score liver parenchymal appearances, PH and bile duct lesions predicted radiological progression from baseline with high accuracy (AUROC, >0.8).^55^ Sixty percent of patients developed evolving changes over ∼4 years, and preliminary data indicate baseline radiological score to be a highly sensitive prognosticator of clinical outcome, with the most predictive components relating to parenchymal as opposed to ductal changes.^56^

### Dominant Strictures

Dominant strictures (DS) were originally defined based on historical ERC findings, and consensus opinion as to how such lesions are to be classified noninvasively is yet to be delivered. Observational studies report a presenting frequency of 12%‐60%,^57^, ^58^ with no population‐level indications of true incidence. Natural history data are similarly restricted to specialist centers, with reduced survival largely reflecting difficulties in CCA recognition.^18^, ^50^, ^58^, ^59^ However, more recent reports suggest actuarial TFS as significantly poorer irrespective of cancer development and heavily influenced by presence of colitis.^50^, ^60^ Several investigators report biochemical and clinical improvements after endoscopic therapy,^61^ but the prognostic impact of intervention needs assessment.

### Small Duct PSC

Small duct PSC (sdPSC) represents 10%‐15% of the disease spectrum, with affected individuals less often symptomatic.^62^ There is now well‐validated evidence that disease progression is relatively infrequent, occurring over a longer time period than the classical form.^13^, ^63^, ^64^ Although colitis manifests to a similar degree there is little to suggest an impact on liver‐related outcomes; and given that survival patterns mirror those of an age‐ and sex‐matched population, the need for investigative therapy is perhaps less perceptible in those with the small duct variant.

## Histological Stage and Noninvasive Evaluation

Disease identification in PBC and PSC is largely reliant on serology and cholangiography, respectively, in the appropriate clinical and biochemical context. Nevertheless, liver biopsy is invaluable in cases of diagnostic doubt and provides key information with regard to disease activity and severity that may improve predictive power of existing algorithms.^31^

Several contemporary histological systems have emerged for PBC,^65^, ^66^ with the aim of accurately representing interface activity, ductopenia, chronic cholestasis, and fibrotic indices—variables well known to forecast biochemical nonresponse and clinical outcome (Table 5).^9^, ^10^, ^14^, ^25^, ^30^, ^31^, ^67^, ^68^ Common histological changes in PSC include interface activity, ductopenia and concentric periductal fibrosis, although individual prognostic weightings are unclear, and no disease‐specific classification exists. Nevertheless, data extrapolated from the Dutch population‐based registry (n = 64) indicate that scoring through PBC‐based classification systems, as well as lobular fibrosis stage (Ishak), significantly associates with time to transplantation in PSC patients.^69^

**Table 5:** Prognosis‐Related Histological Themes in PBC

Feature	Comments
**Fibrosis**	• Advanced septal fibrosis predictive of UDCA failure and clinical outcome^10^, ^25^, ^68^
**Interface hepatitis**	• Positive correlation with AST/ALT (Spearman's *ρ*: 0.469/0.395; *P* < 0.05)^66^ • Moderate‐severe activity^a^ independently predictive of biochemical nonresponse, histological stage progression, progression to transplantation, and death (relative risk: 1.9; *P* = 0.002)^10^, ^30^, ^31^, ^72^ • Improvement in interface activity (in the absence of significant fibrosis) with corticosteroids reported in at least two randomized trials and one multicenter observational study^77^
**Ductopenia**	• Negative correlation of bile duct ratio^b^ with serum ALP (Spearman's *ρ*: −0.362; *P* < 0.05)^66^ • Duct loss in >50% of portal tracts predicts histological disease progression and failure to meet biochemical response^25^ • Premature ductopenic variant affects 5%‐10% of patients: characterized by: rapid‐onset bile duct loss without significant baseline fibrosis, severe icteric cholestasis, and rapid progression toward cirrhosis (<5 years^67^)
**Chronic cholestasis**	• Deposition of orcein‐positive granules in periportal hepatocytes predictive of development of cirrhosis‐related conditions^65^

a Moderate: segmental necrosis at periphery of >50% of portal tracts or circumferential necrosis in <50% of portal tracts; severe: circumferential necrosis in >50% of portal tracts.

b Ratio of the number of portal tracts with ducts to total number of portal tracts.

Histology remains the gold standard for assessing fibrosis progression—a clear determinant of clinical outcome. However, the intrusiveness, coupled with well‐known sampling variability and disconcordant reporting in cholestatic disease, has fostered development of several noninvasive surrogates (Table 6). In the current clinical climate, histological stratification holds limited routine applicability, although staging systems and evaluation of prognosis‐related histological lesions may have a place as surrogate endpoints in clinical trials—a topic beyond the scope of this review.

**Table 6:** Noninvasive Evaluation of Liver Fibrosis

Modality	PBC	PSC
**VCTE**	***Precedents*:** • Increased risk of clinical events (decompensation, LT, and liver‐related mortality) independent of biochemical response in patients with LSM >9.6 kPa, or ΔLSM >2.1 kPa/yr^70^	***Precedents*:** • Higher baseline LSM (>9.9kPa most sensitive), or ΔLSM>1.3kPa/yr. predictive of adverse clinical events.^72^ Low‐risk group best discriminated by LSM ≤6.5kPa
	***Studied cohorts*:** • n = 150; single‐center and UDCA treated	***Studied cohorts*:** • *n* = 167; single‐center
	***Comment*:** • Proven surrogate of fibrosis in PBC. However, validation as an outcome predictor pending	***Comment*:** • Impact of severe cholestasis/cholangitis/IBD activity uncertain • Validation as an outcome predictor pending
	• Unclear whether adds predictive value to biochemical response status	
**ELF**	***Precedents*:** • Significant differences in clinical event rate between score tertiles^73^ • Δ1‐point increase imparts 3‐fold greater risk of liver‐related events	***Precedents*:** • LT‐free survival significantly greater in patients harboring low (9.7 years) vs. high (1.3 years) ELF scores (threshold, 10.6)^74^
	***Studied cohorts*:** • n = 161; multicenter national data extrapolated from a clinical trial of methotrexate and UDCA	***Studied cohorts*:** • n = 167 (derivation) + 138 (validation)
	***Comment*:** • Unclear whether adds predictive value to biochemical response status	***Comment*:** • Internal multicenter validation. However, short disease duration in LT‐free survivors in the original report (<5 years)
	• Impact of longitudinal stability vs. fluctuations over time yet to be determined • Not yet validated	• Impact of longitudinal stability vs. fluctuations over time yet to be determined • Unclear whether stratifier of disease severity vs. stage
	• Unclear whether stratifier of disease severity vs. stage	

Abbreviation: kPa/yr, kilopascals per year.

### Vibration Controlled Transient Elastography

The accuracy of vibration controlled transient elastography (VCTE) in fibrosis staging has been demonstrated in at least two large PBC cohorts,^70^, ^71^ with prognostic capabilities independent of biochemical response evident in a recent single‐center retrospective study of 150 patients.^70^ Though VCTE outperforms APRI as well as several noninvasive surrogates of fibrosis, it remains unclear whether the former confers additive discrimination to biochemical response. The prognostic impact of liver stiffness measurement (LSM) in PSC has also recently been described,^72^ and as with previous descriptors, correlated well with degree of liver fibrosis but performing best at extremes of histological stage (≤F1 and ≥F3). More striking was the observation that increased baseline measurements and rate of change in LSM were strongly and independently linked with PSC‐specific clinical events.^72^

LSM, in addition to reflecting severity of fibrosis, can also be influenced by extrahepatic cholestasis and may not necessarily capture disease facets, such as hepatic necroinflammatory activity, ductopenia and PH. Nevertheless, encouraging data from existing series strongly support VCTE‐derived LSM—absolute values as well as fluctuations over time—as major predictors of AEs. Given correlations with mortality and liver transplantation (LT) in PBC and PSC, VCTE may represent a generic surrogate in chronic cholestatic liver disease, and prospective validations as part of multicenter collaborative efforts continue to emerge.

### Enhanced Liver Fibrosis Score

The enhanced liver fibrosis (ELF) score bears similar prognostic utility to histological fibrosis staging in PBC,^73^ although akin to VCTE, additive predictive value to biochemical response has not been demonstrated. More recent focus on the stratifying properties in PSC led to a notable publication by the Norwegian Study Group. Therein, patients exhibited significantly divergent TFS curves according to tertile distribution, or through a dichotomous Youden‐index‐derived cutpoint.^74^ Moreover, ELF score correlated well with elastography and provided incremental prognostic utility to Mayo risk. However, one caveat is the relatively short disease duration experienced by transplant‐free survivors (median, 0.2 years) and of further uncertainty is how dynamic fluctuations impact outcome longitudinally. Nevertheless, this study represents the first noninvasive, externally validated serum biomarker panel in PSC.

## Clinical Integration and Prospective Outlook

Biochemical nonresponders represent the most readily identifiable at‐risk group in PBC, and incorporating a step‐wise algorithm with response criteria as the central feature is likely to capture the greatest breadth of individuals who will benefit from clinical trials (Fig. 2A). Validation at interim time points for groups who commonly experience treatment failure is urgently decreed and may assist in the earlier identification of high‐risk patients. Along similar lines, prospective banking of biological materials with paired long‐term clinical follow‐up data could yield predictive markers from the point of diagnosis through interrogation of key pathways underlying nonresponse. The few PBC patients who endure AEs despite attainment of response remain poorly defined, but increasingly recognized^28^, ^35^; and the additional impact of “biochemical escape”—wherein previous responders develop subsequent elevations in laboratory parameters—yet to be explored. The additive predictive value of histology and its noninvasive surrogates to existing criteria also requires further validation in a manner similar to that presented for APRI, in addition to newer biochemical response criteria with dynamic predictive capabilities.^31^, ^34^, ^35^

**Figure 2A:**
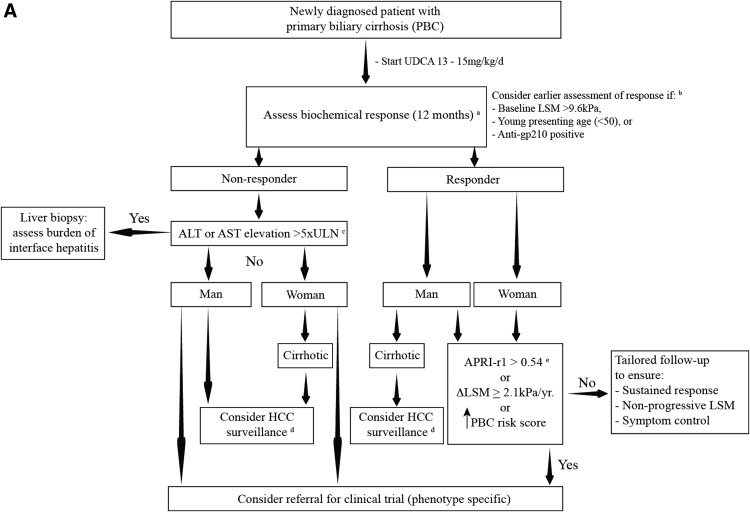
Proposed pathway to clinical trial recruitment in PBC. Biochemical response criteria are the most robust of all predictive modalities, with greatest chance of attainment after UDCA provision. Current strategies require assessment at 12 months (A), although increasing identification of presenting phenotypes in which therapeutic failure is more common may call for earlier application of response criteria (e.g., at 6 months) if validation holds true (B). This group is speculated to include young women, and where available, those who test positive for anti‐gp210 reactivity or who exhibit an elevated baseline LSM as measured by transient elastography. To date, Paris I has been externally validated as the most accurate discriminator (optimal response models may differ according to study population), and certain individuals fail therapy predominantly on transaminase indices (C). Though not necessarily classifying an “overlap syndrome,” significant interface hepatitis may be conducive to adjuvant corticosteroids, and an argument for stratification through liver histology is presented at this stage.^14^, ^68^ Biochemical nonresponse imparts additional HCC risk (D), with highest incidence in patients with cirrhosis; and men irrespective of disease stage. Additionally, some patients experience progressive liver disease despite fulfilling response criteria (E), and sequential application of APRI or newer PBC risk scoring systems (age‐adjusted, UK‐PBC or GLOBE score, that is greater than that present at baseline/PBC diagnosis) can assist in their early recognition. The additional discriminatory value of annual LSM change (once substantiated) may be applied in a similar vein. All patients with evidence suggestive of PH, irrespective of liver disease stage, are also recommended to undergo endoscopic variceal surveillance according to current guidelines and local expertise, given the negative clinical impact of varices on disease outcome.^4^ Incorporating such a step‐wise algorithm to all newly presenting, well‐compensated patients (outside of transplantation criteria) will likely capture the greatest breadth of at‐risk individuals, wherein therapeutic shortfall is most evident. Abbreviation: kPa/yr, kilopascals per year.

By contrast, safe discrimination of risk phenotypes in PSC is not possible through early application of a single modality, and timely assessment requires harnessing multiple predictive techniques collectively (Fig. 2B). Despite invasiveness of histological stratification, the advent of VCTE and related biomarkers hold promise, although predictive performance is best at stages of advanced fibrosis implying surrogacy toward disease stage, rather than severity, and prospective validation currently remaining. Present biochemical surrogates are far from robust, and it is crucial for future endeavors to secure appropriate control groups before stratifying PSC patients as low risk based on serum ALP alone, particularly given that 20% of UDCA‐treated patients with normal laboratory values still develop progressive disease.^38^ Further efforts are also needed to appraise the relative independence of existing parameters that stratify risk, both consequentially and concurrently.

**Figure 2B:**
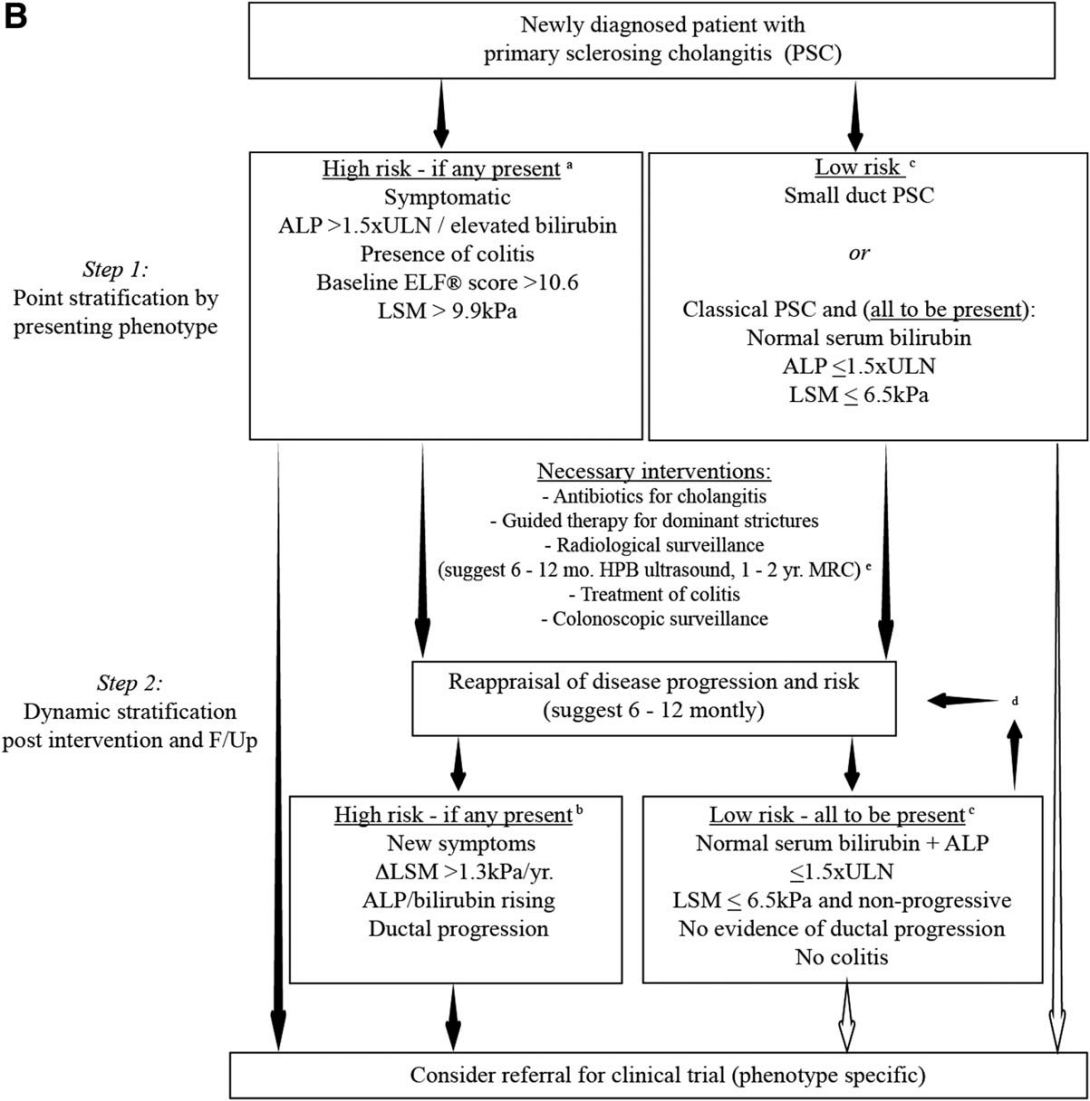
Proposed pathway to clinical trial recruitment in PSC. The unpredictable clinical nature and dearth of effective medical therapy in PSC means that the vast majority of patients (outside of transplant criteria) currently harbor >1 high‐risk classifier at time of presentation (A), including the presence of colitis, persistently elevated liver biochemistry, or features predictive of advancing fibrosis or future cholangiographic progression. Symptomatic presentations, in addition to indicators of advancing fibrosis, also predict adverse clinical outcome, although the relative and independent predictive value between modalities are yet to be established in PSC, with ELF score being somewhat restricted and of limited routine availability. Moreover, as a continuous variable, the optimum stratification threshold utilizing elastography is not yet defined, with LSM >9.9 kPa the best discriminator for identifying high‐risk individuals, yet ≤6.5 kPa most indicative of early disease. Nevertheless, the dynamic impact of chronal increments is well demonstrated for elastography (B) and possibly for progressive MRC scores (not illustrated; formal publication pending); signifying further groups in whom clinical trials should be encouraged. Conversely, asymptomatic patients with small duct disease, as well as those with classical PSC achieving persistently low/normal liver biochemistry who maintain stable fibrotic indices in the absence of cholangiographic progression, likely herald a more consistent low‐risk profile (C), albeit with need for longitudinal appraisal (D) given that early predictive models of disease progression are not yet available. Indeed, regular risk assessment of malignant complications is critical to ensure long‐term patient safety, given that no early or robust predictors of future CCA currently exist. To this effect, a position for even those in the lower‐risk category (with large duct disease) to be considered for clinical trials specifically targeted at reducing CCA incidence can also be argued (open arrows), while accepting the strong probability that other PSC‐related clinical events develop at a low incidence. The optimum frequency of routine radiological surveillance is often debated (E), with no evidence‐based guidance in this regard. A suggested policy of 12‐monthly (detection of gallbladder polyps), or 6‐monthly in patients with cirrhosis (HCC surveillance) is proposed in keeping with current guidelines. Abbreviations: F/Up, follow‐up; HPB, hepatopancreatobiliary; kPa, kilopascals.

## Conclusion

Patients with PBC and PSC remain a heterogeneous cohort with concerns surrounding reliable outcome forecasting. Stratification paradigms are shifting with increased efforts toward recognition of at‐risk phenotypes. The increased utilization of such tools, both clinically and in trial settings, is hoped to allow for more personalized care. In so doing, low‐risk patients can be reassured and managed accordingly, whereas higher‐risk individuals are offered tailored care, as well as access to carefully designed trials relevant to their disease course.

Author names in bold designate shared co‐first authorship.

## Supporting information

Additional Supporting Information may be found in the online version of this article at http://onlinelibrary.wiley.com/doi/10.1002/hep.28128/suppinfo.

Supporting Information
