# Supplementary material for: Risk stratification in autoimmune cholestatic liver diseases: Opportunities for clinicians and trialists
Source: Hepatology. 2015 Nov 26;63(2):644–59. doi: 10.1002/hep.28128 (PMC4864755; doi:10.1002/hep.28128)
Supplement: Supplementary file 1 — Supporting Information [file HEP-63-644-s001.pdf]

Supplementary Table 1: GRADE criteria for assessment of prognosis (78)

Quality level	Description	GRADE Score
High	We are very confident that the true prognosis (probability of future events) lies close to that of the estimate	A
Intermediate	We are moderately confident that the true prognosis (probability of future events) is likely to be close to the estimate, but there is a possibility that it is substantially different	B
Low	Our confidence in the estimate is limited: the true prognosis (probability of future events) may be substantially different from the estimate	C
Very low	We have very little confidence in the estimate: the true prognosis (probability of future events) is likely to be substantially different from the estimate	D
